# Edible Insects as a Next-Generation Protein Platform for Sustainable Food Systems

**DOI:** 10.3390/foods15132362

**Published:** 2026-07-02

**Authors:** Annalaura Brai, Elena Dreassi

**Affiliations:** Department of Biotechnology, Chemistry and Pharmacy, University of Siena, via A. Moro, 53100 Siena, Italy; elena.dreassi@unisi.it

## 1. Introduction

The global food system is facing multiple, converging pressures. Climate change, biodiversity loss, land degradation, and increasingly volatile geopolitical dynamics are converging to expose the fragility of conventional protein supply chains [1,2,3]. Volatility in trade flows, fertilizer supply, energy markets, and feed logistics has highlighted that protein security is no longer only an environmental concern but a question of systemic resilience [4,5,6].

Animal agriculture represents a key structural vulnerability within this system. While it remains fundamental for global nutrition, its reliance on feed crops, land expansion, and energy-intensive supply chains places it under simultaneous environmental and geopolitical pressures [7]. Against this backdrop, the “protein transition” is no longer an abstract sustainability concept, but a structural requirement for future food security [6].

Edible insects are emerging as one of the few protein sources that directly address this dual challenge. Their relevance extends beyond nutritional composition, although they provide high-quality protein and lipids and essential amino acids [8]. Their key advantage lies in system efficiency: insects convert low-value organic substrates into edible biomass with minimal land, water, and external input requirements, enabling production models that are inherently less exposed to global supply shocks [9,10].

This efficiency aligns closely with circular bioeconomy principles. Insect production can valorize agricultural residues and food industry by-products, thereby reducing dependence and partially decoupling protein generation from conventional feed-food competition [10]. In a context of increasing input scarcity and supply chain fragmentation, this localized and modular production logic becomes strategically relevant.

At the same time, insects are gaining attention as sources of functional bioactive compounds, including peptides, lipids, and chitin-derived biopolymers, with emerging applications in metabolic health and functional food development [9,11]. This broadens their role from alternative protein sources to multi-functional biological platforms for next-generation food systems.

Technological advances in fractionation, protein isolation, and formulation are accelerating the integration of insect-derived ingredients into food products, including hybrid meats and functional formulations [12,13]. Despite this progress, large-scale adoption remains limited. The barriers are now predominantly systemic. Regulatory fragmentation, lack of standardization, and cultural resistance continue to constrain market development [10]. Increasingly, geopolitical instability is adding urgency to the discussion, highlighting the need for protein systems that are more decentralized, resource-efficient, and less dependent on fragile global supply chains [1].

This Special Issue, “Edible Insects as a Healthy and Sustainable Strategy to Handle Protein Demand and Food Insecurity”, addresses this transition at its core. It moves beyond technological validation toward system-level implementation. In doing so, it positions edible insects not as an emerging alternative, but as a structurally relevant component of resilient future protein systems.

## 2. Overview

This Special Issue brings together contributions that collectively map the transition of edible insects from experimental substrates to functional food system components, spanning production optimization, ingredient engineering, product formulation, and socio-cultural acceptance. Within this evolving framework, recent studies further consolidate the view of insects not as alternatives, but as engineered biological systems embedded in future food architectures.

Several studies focus on upstream modulation of insect biomass quality, demonstrating that insect nutritional composition is not fixed but highly responsive to feed strategies and rearing conditions. In particular, Dalle Zotte et al. (Contribution 1) show that dietary manipulation can significantly alter antioxidant capacity, amino acid profiles, and oxidative stability in *Tenebrio molitor* larvae, reinforcing the concept of insects as controllable bioconversion systems rather than static raw materials. Extending this perspective, Chaipoot et al. (Contribution 2) demonstrate that even alternative insect matrices such as honey bee brood can be effectively transformed into stable protein powders via foam-mat drying, where binder systems (CMC and glycerol monostearate) modulate yield and functional properties, highlighting the sensitivity of insect-derived ingredients to formulation design as much as to biological origin.

Complementing this, Verheyen et al. (Contribution 3) provide critical insights into how post-harvest processing variables shape protein extractability and compositional integrity. Their findings highlight a key industrial reality: the scalability of insect protein is not only dependent on farming efficiency but also on downstream processing standardization. In parallel, Alhasyani et al. (Contribution 4) expand this processing dimension toward biofunctional valorization, showing how *Hermetia illucens*–derived bioactives, particularly chitooligosaccharides and peptides, can be directed through enzymatic and thermal pathways to generate gut-health–relevant compounds, while also emphasizing the current gaps in clinical validation and industrial upscaling.

A second cluster of contributions addresses the integration of insect ingredients into conventional food matrices, a necessary step for consumer-driven adoption. Carvalho et al. (Contribution 5) and Hospital et al. (Contribution 6) demonstrate that partial replacement of meat with insect-derived powders can yield hybrid or reformulated products with acceptable technological and sensory properties. These studies collectively support the hypothesis that the most viable near-term pathway for edible insects is not whole-insect consumption, but ingredient invisibility through formulation engineering.

From a systems perspective, Brai et al. (Contribution 7) extend the discussion into circularity, showing how agro-industrial by-products can be valorized through insect bioconversion into high-value protein biomass. This reinforces the role of insects as enabling agents within circular food economy models, rather than as isolated production systems. In a complementary line, Lisboa et al. (Contribution 8) further contextualize this transition by framing insect proteins as multi-dimensional sustainability assets, linking reduced environmental burden with nutrient density, feed conversion efficiency, and system-level resource recovery.

Finally, Guiné et al. (Contribution 9) address the most persistent bottleneck in the field: consumer acceptance. Their multi-country analysis confirms that cultural context remains the dominant determinant of willingness to consume insects, underscoring that technological feasibility alone is insufficient to guarantee adoption. Collectively, these contributions suggest that the trajectory of edible insects will be determined not only by advances in bioprocessing and formulation, but by the co-evolution of technological, regulatory, and cultural systems.

## 3. Conclusions

The papers presented in this Special Issue collectively redefine edible insects as a multi-dimensional solution to protein system transformation. Rather than functioning solely as an alternative protein source, insects emerge as adaptable bioconversion systems, functional ingredient platforms, and potential enablers of circular food economies. However, the transition from scientific potential to systemic adoption remains incomplete. The decisive barriers are no longer related to production feasibility, but to integration: regulatory harmonization, industrial scaling, sensory optimization, and, critically, consumer normalization.

In this context, edible insects should be understood not as an endpoint solution, but as a transitional and transformative technology within the broader restructuring of global protein systems. The research collected here provides a foundation for this transition and highlights the interdisciplinary effort required to move from experimental validation to societal implementation.

## Data Availability

Not applicable.
